# Short Heat Treatments for the F357 Aluminum Alloy Processed by Laser Powder Bed Fusion

**DOI:** 10.3390/ma14206157

**Published:** 2021-10-17

**Authors:** Matteo Vanzetti, Enrico Virgillito, Alberta Aversa, Diego Manfredi, Federica Bondioli, Mariangela Lombardi, Paolo Fino

**Affiliations:** 1 Department of Applied Science and Technology (DISAT), Politecnico di Torino, Corso Duca degli Abruzzi 24, 10129 Torino, Italy; enrico.virgillito@polito.it (E.V.); alberta.aversa@polito.it (A.A.); diego.manfredi@polito.it (D.M.); federica.bondioli@polito.it (F.B.); mariangela.lombardi@polito.it (M.L.); paolo.fino@polito.it (P.F.); 2 Center for Sustainable Future Technologies IIT@Polito, Istituto Italiano di Tecnologia, Via Livorno 60, 10124 Torino, Italy; 3 Consorzio Interuniversitario Nazionale per la Scienza e Tecnologia dei Materiali (INSTM), Via G. Giusti 9, 50121 Firenze, Italy

**Keywords:** additive manufacturing, laser powder bed fusion, aluminum, f357, heat treatments, microstructure, mechanical properties

## Abstract

Conventionally processed precipitation hardening aluminum alloys are generally treated with T6 heat treatments which are time-consuming and generally optimized for conventionally processed microstructures. Alternatively, parts produced by laser powder bed fusion (L-PBF) are characterized by unique microstructures made of very fine and metastable phases. These peculiar features require specifically optimized heat treatments. This work evaluates the effects of a short T6 heat treatment on L-PBF AlSi7Mg samples. The samples underwent a solution step of 15 min at 540 °C followed by water quenching and subsequently by an artificial aging at 170 °C for 2–8 h. The heat treated samples were characterized from a microstructural and mechanical point of view and compared with both as-built and direct aging (DA) treated samples. The results show that a 15 min solution treatment at 540 °C allows the dissolution of the very fine phases obtained during the L-PBF process; the subsequent heat treatment at 170 °C for 6 h makes it possible to obtain slightly lower tensile properties compared to those of the standard T6. With respect to the DA samples, higher elongation was achieved. These results show that this heat treatment can be of great benefit for the industry.

## 1. Introduction

Laser powder bed fusion (L-PBF) can be used to process a wide variety of metallic materials such as steel, titanium, nickel, and aluminum alloys. Aluminum alloys are very interesting for various applications owing to their relatively low cost, light weight, and high strength [1,2,3,4,5]. Among aluminum alloys, traditional cast Al–Si–Mg alloys are the most used and studied ones for the L-PBF technique due to their good laser processability. In particular, AlSi7Mg has attracted by far the greatest interest due to both its nearly eutectic composition that ensures a narrow solidification range and the high Si content that assures a good fluidity in the molten state. These are very important aspects in the L-PBF process as it makes it possible to reach full dense and crack-free parts [6,7,8]. The large interest in the AlSi7Mg alloy is proved by the large number of scientific papers available in the literature [9,10,11,12,13,14,15,16]. This alloy is also generally called A357 (AMS 4219 standard) or F357 (AMS 4289 standard) depending on the beryllium content, which is lower than 0.002% in the F357 standard. Small quantities of beryllium in the alloy change the precipitates’ shape and dimension, and it is therefore important to strictly control its content to optimize the mechanical properties [17]. In particular, the presence of beryllium in the alloy improves its hardness, tensile properties, and corrosion resistance, and reduces the fatigue crack propagation [17,18]. The use of F357 alloy, however, causes a safety problem due to the presence of beryllium, which is classified as a category I carcinogen [17]. Much research in recent years has focused on post-processing heat treatments of L-PBF AlSi7Mg parts generally performed following the thermal treatments used after traditional processing techniques [3,9,11,19,20,21]. The main goals of these treatments are to reduce residual stresses and to modify the microstructure and thus the mechanical properties of samples in the as-built (AB) condition.

Multiple authors have investigated the effect of the standard T6 heat treatment on the microstructure and mechanical properties of L-PBF AlSi7Mg samples [11,12,13,22]. The conventional T6 heat treatment of AlSi7Mg consists of a solution heat treatment (SHT) at 530–550 °C for 6–12 h followed by water quenching and artificial aging at 150–225 °C for 3–6 h [14,15]. The main results were that, after the T6 heat treatment, the yield strength (YS) and elongation at failure (ε) values were comparable to the as-built ones while the ultimate tensile strength (UTS) values lowered [11]. These results are due to the long solution heat treatment performed at high temperatures that causes the loss of the fine microstructure and the consequent growth of coarse Si particles. The subsequent artificial aging allows the precipitation of a fine Mg_2_Si strengthening phase [23]. However, the solution treatment time, as underlined by Sjölander et al. [24], is strongly related to the dimension of the starting microstructural features and the T6 treatments were optimized for the casting microstructure which strongly differs from the additive manufacturing (AM) one.

Recent studies have demonstrated that L-PBF parts could benefit from different heat treatments designed to exploit the unique features of the as-built microstructure [9,25]. One of the most studied post-processing heat treatments in the AM field is direct aging (DA), or T5, which gives the best results in terms of mechanical properties with short times and thus lower costs [2,3,9,12,26]. This heat treatment, which consists of an artificial aging of the AB samples at 160–180 °C for 2–8 h, takes advantage of the supersaturated solid solution of AM samples obtained because of the rapid cooling [3,9,12]. The DA allows supersaturated Si and Mg to precipitate, forming a Mg_2_Si phase and maintaining the L-PBF fine microstructure, leading to high UTS and YS values. Unfortunately, high residual stresses and the fine microstructural features lead to low elongation at failure values [9].

Based on these considerations, it is clear that the heat treatments for AlSi7Mg alloys processed by L-PBF still need to be studied and optimized. In fact, it is important to find a short heat treatment able to guarantee a fine microstructure, good mechanical properties, and low residual stresses while reducing cost and lead time. Therefore, the purpose of this work is to evaluate the effects of a short heat treatment L-PBF AlSi7Mg samples, characterized by a solution heat treatment of 15 min followed by water quenching and subsequently by an artificial aging at 170 °C for 2–8 h. With this aim, the mechanical properties and the microstructure of the obtained samples were studied in comparison with as-built and direct-aged conditions.

## 2. Materials and Methods

An F357 gas atomized powder, supplied by Valimet Inc. (Stockton, CA, USA), with the chemical composition reported in Table 1, was used to produce the samples.

**Figure 1:**
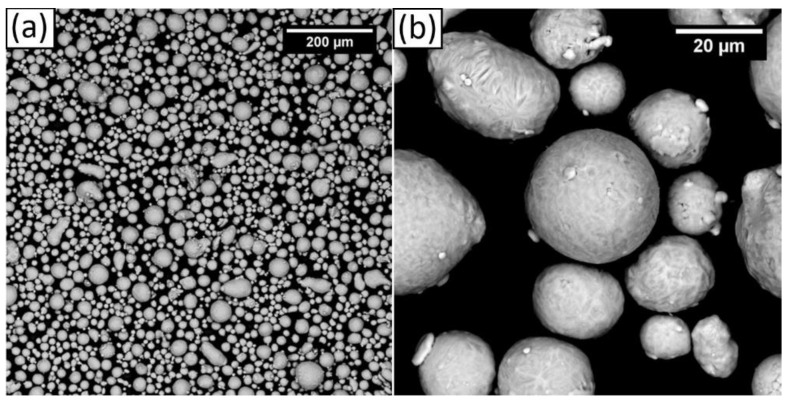
F357 powder at (**a**) 300× and (**b**) 3000×.

The scanning electron microscope (SEM) images, reported in Figure 1, show that most of the particles had a quasi-spherical shape, and the remaining particles had an elongated shape. The d10, d50, and d90 were 17, 34, and 60 µm, respectively. These values are in line with those generally used for the L-PBF process of aluminum alloys with 30 µm layer thickness [26].

All samples were built using an EOS M270 Dual Mode system (EOS GmbH, Krailling/Munich, Germany) which uses an ytterbium fiber laser with a maximum power of 200 W in argon atmosphere using the EOS 67° rotated stripe scanning strategy.

For the first job, the building parameters were optimized using a laser power of 195 W and a layer thickness of 30 µm, varying both laser scan speed (700, 800, and 900 mm/s) and hatching distance (0.12, 0.15, and 0.17 mm). Two cubic samples for each combination of parameters were produced [26]. These building parameters were chosen based on the processing data of similar alloys [3,11]. To optimize the building parameters, the density of each sample was evaluated by Archimedes’ method after a mild polishing of the surface. The building parameters of the densest sample were chosen, among the tested parameters, for the second job.

In the second job, cubic samples with 10 mm size and 16 cylindric samples with a length and diameter of 140 and 14 mm, respectively, for tensile tests were produced using the optimized parameters. Two different types of heat treatment were performed on the as-built cubes. The direct aging (DA) was performed at 170 °C for a time period ranging from 2 to 8 h [7], while the short T6 proposed treatment had a short solution heat treatment (S-SHT) at 540 °C for 15 min followed by a water quenching step and a final aging treatment at 170 °C for a time period ranging from 2 to 8 h [27]. The S-SHT length was selected based on the work of Sjölander et al., who indicated that 10 min is a sufficient time to achieve dissolution and homogenization for a very fine microstructure [24]. In Table 2, the details of the performed heat treatments and the used sample codes are reported.

As-built and heat treated cubic samples were cut parallel to the Z axis, i.e., the building direction, polished to 0.03 µm, and etched with a Keller solution for 10 s. In order to preliminarily assess mechanical properties, hardness measurements were performed on sample cross-sections using a micro Vickers indenter (Leica VMHT, Leica, Wetzlar, Germany) with a load of 100 g applied for 15 s. Ten indentions were performed for every cross-section. To analyze the microstructure, cross-sections were observed through a Leica DMI 5000 M (Leica, Wetzlar, Germany) optical microscope and a Tescan S9000G SEM (Tescan Company, Brno, Czech Republic). To evaluate the precipitated phases, X-ray diffraction (XRD) measurements were performed on the cubes’ X-Z cross-section using an X’Pert Philips diffractometer (PANalytical, Almelo, The Netherland) in a Bragg–Brentano configuration in a 2θ range between 20° and 100° θ, with a step size of 0.013° and 30 s per step. The precipitation phenomena were studied by means of a Differential Scanning Calorimeter (DSC, Setaram TGA 92 16.18, Setaram Instrumentation, Caluire, France). The analyses were performed in Ar atmosphere using 20 °C/min as a heating ramp from 25 °C to 540 °C. Tensile samples, with a length and diameter of 40 and 8 mm, respectively, were machined from the cylindrical samples according to the ASTM E8 standard. They were then tested by a ZwickRoell ProLine Z0505 tensile machine (ZwickRoell GmbH & Co. KG, Ulm, Germany) using an 8 × 10^−3^ s^−1^ strain rate. Three repetitions were performed for each selected heat treatment.

## 3. Results and Discussion

### 3.1. Optimization of Process Parameters

**Figure 2:**
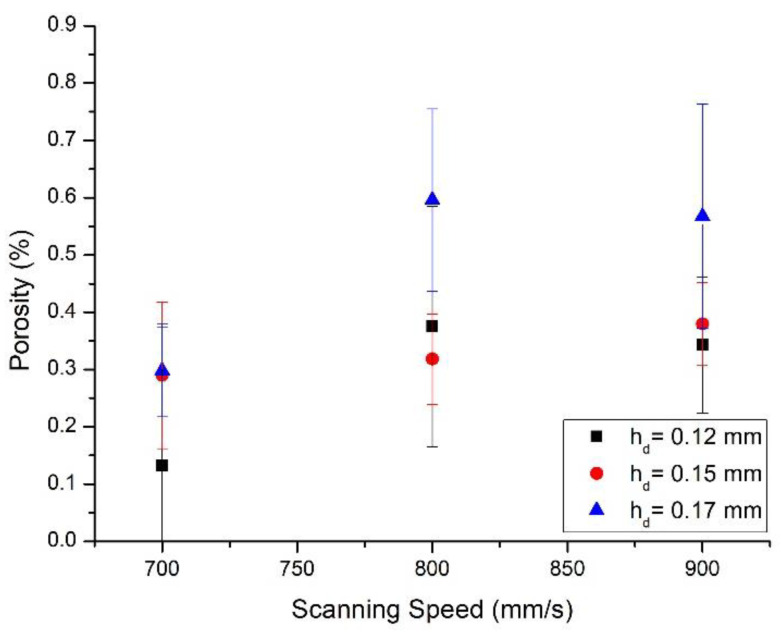
Porosity versus scanning speed graph for the F357 powder processed using 30 µm as layer thickness, 195 W as laser power, and varying hatching distance.

To assess the best combination of process parameters to produce full dense F357 parts, the sample porosity was studied by varying the scanning speed (v) and the hatching distance (h_d_). The evolution of the porosity as a function of building parameters is reported in Figure 2. It can be noticed that most of the parameter sets lead to low porosity values (i.e., <1%). The analysis of the data reveals that lower scanning speed and hatching distance results in lower porosity. Considering the selected power of 195 W, it is clear from this graph that the optimal combination of parameters among the tested ones is v = 700 mm/s and h_d_ = 0.12 mm. These parameters were used to produce all the samples of the second job.

### 3.2. Hardness

**Figure 3:**
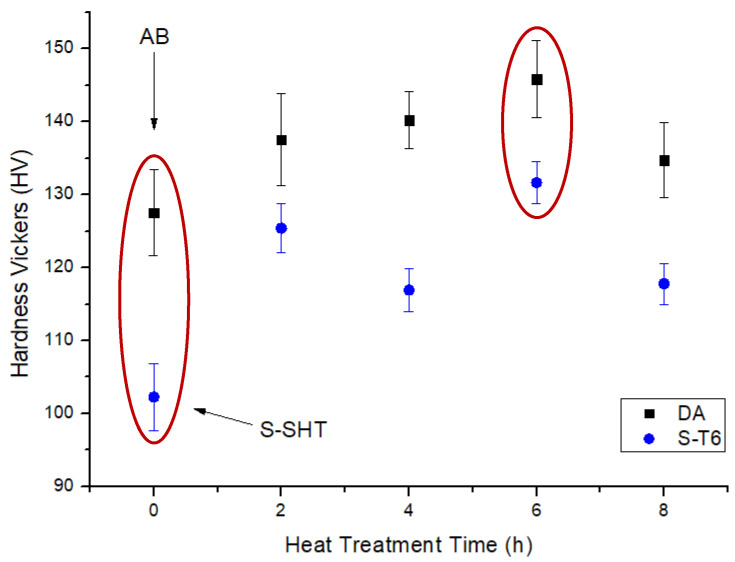
Hardness trends during DA and S-T6 aging at 170 °C. The red circles highlight the selected heat treatments.

The hardness comparison of the F357 samples in the as-built (AB) and heat treated conditions is shown in Figure 3. The AB sample presented a hardness value of 127 ± 6 HV; similar results were obtained in previous works [3,19,20]. The sample hardness increased as a consequence of the DA heat treatment performed at 170 °C, reaching the highest value of 146 ± 5 HV after 6 h (DA-6). This trend was observed in many other studies [10,20]. The S-SHT sample had the lowest hardness value of all the tested samples (102 ± 5 HV). The sample hardness increased because of the aging treatment with a behavior similar to that of the DA samples. The highest S-T6 hardness value was 132 ± 3 HV after a 6 h aging treatment (S-T6-6). The hardness values evaluated in this study were slightly higher compared with the corresponding hardness values measured by Casati et al. with a lower aging temperature of 160 °C [12].

After these considerations, AB, DA-6, S-SHT, and S-T6-6 conditions (highlighted by two red circles on the graph in Figure 3) were selected for the further analyses to understand the relationship between material microstructure and properties.

### 3.3. XRD Analysis

**Figure 4:**
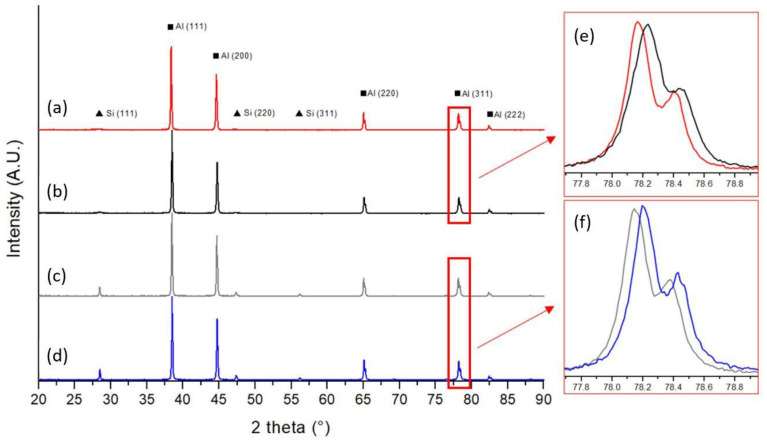
XRD patterns of (**a**) AB, (**b**) DA-6, (**c**) S-SHT, and (**d**) S-T6-6 samples and inset of (**e**) AB and DA-6 and (**f**) SSHT and S-T6-6 samples.

In Figure 4, the XRD patterns of the previously selected conditions are reported. AB (Figure 4a) and DA-6 (Figure 4b) conditions present similar XRD patterns with rather wide and low intensity Si peaks as a consequence of the presence of fine Si particles in the Al matrix. In the DA-6 condition, these peaks are slightly higher in intensity because of the partial precipitation of Si, which was in a supersaturated solid solution in the as-built sample. After S-SHT (Figure 4c) and S-T6-6 (Figure 4d) heat treatment, Si peaks are clearly visible that are relatively high in intensity and narrowed as result of enlarged crystallite size [9]. Additionally, it can be noted that, in these cases, the peaks at about 45°, due to the reflection of the (200) α-Al phase, are slightly higher in intensity than the AB and DA-6 ones, probably due to a partial Si recrystallization after the solution heat treatment. A closer look to the (311) peaks at about 78.2° reveals that in the DA-6 (Figure 4e) and S-T6-6 (Figure 4f) samples, the peaks are shifted to higher 2θ angle values with respect to those of AB and S-SHT, respectively. This shift could be attributed to Si and Mg_2_Si precipitation from the supersaturated solid solution, which leads to a lattice contraction and to the relaxation of residual stresses as studied by Zhang et al. [28,29]. In all cases, the Mg_2_Si strengthening phase was not detected by XRD analysis probably because of the relatively low quantity compared to the X-ray detection limit.

### 3.4. DSC Analysis

**Figure 5:**
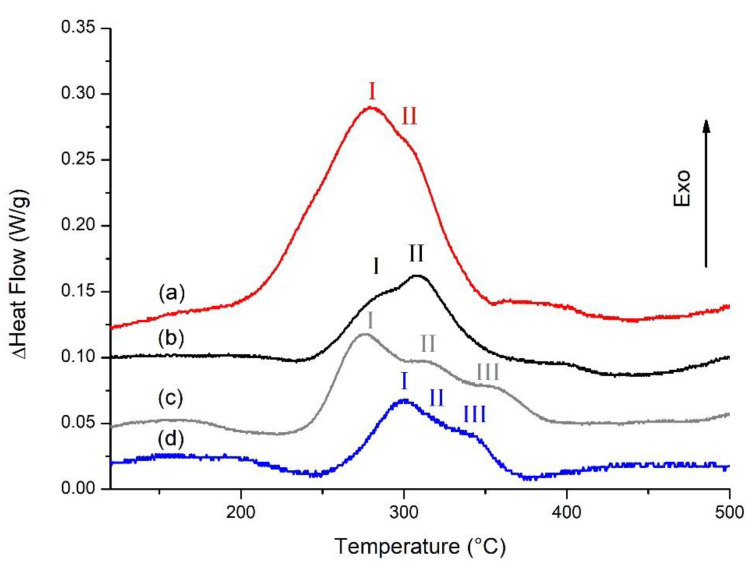
DSC curves of F357 samples in the (**a**) AB, (**b**) DA-6, (**c**) S-SHT, and (**d**) S-T6-6 conditions. Peaks I, II, and III are referred to as Si, Mg_2_Si, and iron phases precipitation, respectively.

The results of the DSC tests, illustrated in Figure 5, allow for the comparison of the phase evolution in the as-built and heat treated samples. It can be noted that the curve of the AB sample (Figure 5a) has two exothermal peaks: the highest in intensity at about 270 °C (I) can be attributed to the precipitation of Si and the second, at about 300 °C (II), can be attributed to the precipitation of Mg_2_Si as reported by Marola et al. for AlSi10Mg [2,28]. The DA-6 curve (Figure 5b) is comparable with the AB one with the first peak being significantly lower in intensity. This behavior, supported by the XRD results, can emphasize that the precipitation of Si occurred during artificial aging. On the other side, the S-SHT curve (Figure 5c) presents three different exothermal peaks at about 270 °C (I), 300 °C (II), and 350 °C (III). As for the AB curve, the first two peaks can be attributed to the precipitation of Si and Mg_2_Si, while the third one could be owed to iron phases, as reported by Casati et al. [12]. The first peak is lower in intensity than the corresponding one in the AB curve due to the partial Si particles precipitation during the solution heat treatment. The S-T6-6 curve is similar to the S-SHT curve with the first peak being lower owing to the artificial aging that S-T6-6 underwent. Moreover, the S-T6-6 peaks were shifted to higher temperatures with respect to the correspondent peaks on the S-SHT curve; this confirms that precipitation phenomena occurred during the aging treatment, as reported by Liu et al. [30]. In fact, the precipitation of Mg–Si clusters reduces the degree of super saturation of the solid solution and decreases the driving force for cluster nucleation, leading to a shift of the DSC peaks of precipitates toward higher temperatures.

### 3.5. Microstructure

**Figure 6:**
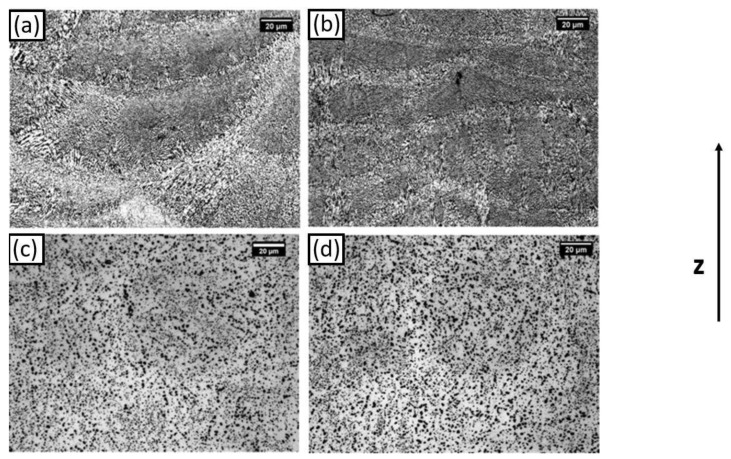
Optical micrographs at 500 × of (**a**) AB, (**b**) DA-6, (**c**) S-SHT, and (**d**) S-T6-6 F357 samples.

**Figure 7:**
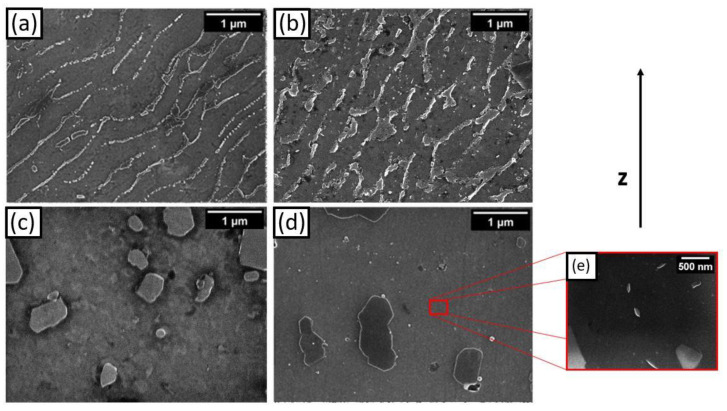
SEM micrographs at 60 k× for (**a**) AB, (**b**) DA-6, (**c**) S-SHT, (**d**) and S-T6-6 F357 samples, and at 100 k× for (**e**) small precipitates in the Al matrix.

Figure 6 and Figure 7 show the optical and SEM micrographs of the AB, DA-6, S-SHT, and S-T6-6 samples. The microstructures of the AB and DA-6 samples (Figure 6a,b) were composed of melt pools produced by the laser during the layer-by-layer melting process, a typical L-PBF microstructure observed in many studies [9,11,21]. SEM micrographs show at higher magnification that the AB sample microstructure (Figure 7a) was constituted of cellular α-Al surrounded by a eutectic Si network due to the rapid cooling and solidification. The DA-6 condition microstructure (Figure 7b) was very similar to the AB ones but with a partially broken eutectic Si network and a small quantity of round and elongated particles smaller than 1 µm in the α-Al cells. With regard to the S-SHT and S-T6-6 samples (Figure 6c,d), the melt pools vanished, showing round micrometric Si particles homogenously distributed in the α-Al matrix. High magnification SEM images show that as a consequence of the S-SHT and S-T6-6 heat treatment (Figure 7c,d), the eutectic Si network completely disappeared and coarsened Si particles with an irregular shape were formed. A similar microstructure with smaller Si dimensions (about 1–2 µm) was observed by Rao et al. in an L-PBF sample after a standard T6 treatment [22]. The reduced Si size observed in the S-SHT and S-T6-6 samples was probably caused by the shorter solution heat treatment. Furthermore, small precipitates and Fe-rich particles can be seen in the Al matrix of the S-T6-6 sample due to the artificial aging that occurred (Figure 7e), as reported by Casati et al. [12]. These findings are in good agreement with the previous XRD and DSC analysis and allow for an understanding of the hardness values (Figure 3). In fact, due to its very fine microstructure, the AB sample was characterized by high hardness values, while the increase in hardness of the DA-6 samples was attributed to the precipitation of Si and Mg_2_Si phases. The lowest hardness value of the S-SHT sample was due to the different microstructure where the interconnected Si network that characterized the AB sample was transformed into a dispersion of Si particles with irregular shape. Furthermore, as for the DA-6 samples, the S-T6-6 sample hardness also increased due to the precipitation of the strengthening phases.

### 3.6. Mechanical Properties

**Figure 8:**
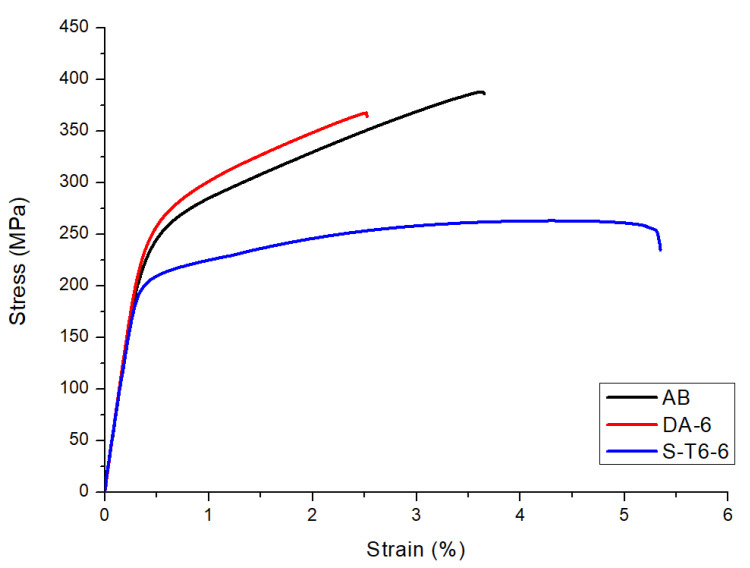
Representative tensile curves of L-PBF F357 samples in AB, DA-6, and S-T6-6 conditions.

In Figure 8, the most representative tensile curves of the L-PBF F357 samples in different conditions are reported. As expected, the DA-6 sample had the highest YS with a value of 268 MPa, but with a reduced elongation at break with respect to the AB sample. This result confirms that the precipitation of the phases that arose due to the DA allowed for the strengthening of the material but reduces its elongation at break. The S-T6-6 treatment showed considerably higher elongation at break than the other two conditions with a mean value of 4.5% and with a slightly lower YS and UTS.

The mechanical properties of the samples studied in this work are summarized in Table 3 together with other literature data of both A357 and F357 alloys. From the data analysis, it can be noticed that, starting from almost the same AB properties [11], the proposed S-T6-6 treatment allowed slightly lower tensile properties to be obtained with respect to traditional T6 treatments performed on L-PBF AlSi7Mg samples. This indicates that the shorter solution treatments could be promising for L-PBF parts because, with the same mechanical properties, they could be strongly beneficial in terms of part production time and thus costs.

## 4. Conclusions

In this work, building parameters to produce fully dense F357 samples by the L-PBF AM technique were optimized, and two types of heat treatments were studied: direct aging and short T6 heat treatments. As-built and heat treated F357 samples were analyzed in terms of microstructure and mechanical properties and the results can be summarized as follows:

- The F357 powder can be easily processed by L-PBF. The optimized parameters to produce F357 fully dense samples were P = 195 W; v = 700 mm/s; h_d_ = 0.12 mm.
- The F357 sample hardness increased after direct aging compared to the as-built condition. A 6 h DA heat treatment at 170 °C led to the highest hardness value of 146 ± 5 HV. The S-SHT sample was characterized by the lowest hardness value because of the dissolution of the alloying elements and the subsequent aging heat treatment at 170 °C caused the hardness to increase up to 132 ± 3 HV.
- The microstructures of AB and DA-6 samples were composed of melt pools and cellular α-Al surrounded by a eutectic Si network which was partially broken after 6 h DA treatment. Furthermore, the α-Al cells of the DA-6 samples contained some fine Si precipitates.
- The microstructures of S-SHT and S-T6-6 were homogenous, did not present melt pools, and were characterized by the presence of Si particles of about 1–2 µm mean size with irregular shape.
- The tensile tests revealed that the DA-6 sample presented the highest YS with a value of 268 MPa but with a reduction in elongation at break with respect to the AB sample. The S-T6-6 treatment showed considerably higher elongation at break than the other two conditions, with a slightly lower YS and UTS.

These results indicate that the suggested short T6 heat treatment is promising for L-PBF F357 samples. The microstructural and mechanical characterizations confirmed that the 15 min solution heat treatment allows for the obtainment of a supersaturated solid solution in a shorter time with respect to the conventional SHT of about 8 h. The subsequent T6 treatment leads to an increase of the hardness value. The use of this heat treatment could be strongly beneficial for the production line as it would imply a reduction of the post processing time and the cost of AM parts.

## Figures and Tables

**Table 1:** Nominal composition of the F357 powder.

	Si	Mg	Ti	Fe	Cu	Zn	Mn	Be
wt%	6.5–7.5	0.45–0.60	0.20	0.15	0.05	0.05	0.03	<0.002

**Table 2:** Details of the investigated heat treatments and sample codes. ^1^ AC = Air Cooling.

Heat Treatment	Heat Treatment Procedure	Sample Code
Direct Aging	170 °C/2 h/AC ^1^	DA-2
	170 °C/4 h/AC	DA-4
	170 °C/6 h/AC	DA-6
	170 °C/8 h/AC	DA-8
Short Solution Heat Treatment	540 °C/15 min/Water Quench	S-SHT
Short T6	S-SHT/170 °C/2 h/AC	S-T6-2
	S-SHT/170 °C/4 h/AC	S-T6-4
	S-SHT/170 °C/6 h/AC	S-T6-6
	S-SHT/170 °C/8 h/AC	S-T6-8

**Table 3:** Tensile properties of F357 and A357 produced by L-PBF and conventional processing.

Alloy	Heat Treatment	YS (MPa)	UTS (MPa)	ε (%)	Ref.
F357	AB	254 (0.05)	382 (6)	3.5 (0.2)	This work
F357	DA-6	268 (0.6)	362 (0.6)	2.4 (0.2)	This work
F357	S-T6-6	218 (21)	270 (18)	4.5 (0.8)	This work
A357 L-PBF	AB	245 (5)	389 (5)	5.2 (0.4)	[11]
A357 L-PBF	T6	250 (10)	308 (12)	5.2 (0.2)	[11]
A357 L-PBF	DA	308 (3)	400 (9)	3.9 (0.7)	[12]
A357 L-PBF	T6	256 (4)	306 (5)	4.7 (0.1)	[12]
A357 L-PBF	SR T6	249 (9)	307 (10)	5.1 (0.3)	[20]
A357 cast	T6	281 (2)	305 (3)	4.1 (0.1)	[31]

## Data Availability

The data presented in this study are available on request from the corresponding author.
